# Characterization of the Structural Requirements for the NADase Activity of Bacterial Toll/IL-1R domains in a Course-based Undergraduate Research Experience

**DOI:** 10.4049/immunohorizons.2300062

**Published:** 2024-08-22

**Authors:** Tamara Vallejo-Schmidt, Cheyenne Palm, Trinity Obiorah, Abra Rachida Koudjra, Katrina Schmidt, Alexis H. Scudder, Eber Guzman-Cruz, Lenora Paige Ingram, Britney C. Erickson, Victoria Akingbehin, Terra Riddick, Sarah Hamilton, Tahreem Riaz, Zachary Alexander, Jasmine T. Anderson, Charlotte Bader, Phoebe H. Calkins, Sumra S. Chaudhry, Haley Collins, Maimunah Conteh, Tope A. Dada, Jaira David, Daniel Fallah, Raquel De Leon, Rachel Duff, Itohan R. Eromosele, Jaliyl K. Jones, Nastaran Keshmiri, Mark A. Mercanti, Jaine Onwezi-Nwugwo, Michael A. Ojo, Emily R. Pascoe, Ariana M. Poteat, Sarah E. Price, Dalton Riedlbauer, Louis T. A. Rolle, Payton Shoemaker, Alanna Stefano, Michaela K. Sterling, Samina Sultana, Lindsey Toneygay, Alexa N. Williams, Sheeram Nallar, John E. Weldon, Greg A. Snyder, Michelle L. D. Snyder

**Affiliations:** * Department of Biological Sciences, Towson University, Towson, MD; † Division of Vaccine Research, Institute of Human Virology, Department of Microbiology and Immunology, University of Maryland, School of Medicine, Baltimore, MD

## Abstract

TLRs initiate innate immune signaling pathways via Toll/IL-1R (TIR) domains on their cytoplasmic tails. Various bacterial species also express TIR domain-containing proteins that contribute to bacterial evasion of the innate immune system. Bacterial TIR domains, along with the mammalian sterile α and TIR motif-containing protein 1 and TIRs from plants, also have been found to exhibit NADase activity. Initial X-ray crystallographic studies of the bacterial TIR from *Acinetobacter baumannii* provided insight into bacterial TIR structure but were unsuccessful in cocrystallization with the NAD^+^ ligand, leading to further questions about the TIR NAD binding site. In this study, we designed a Course-Based Undergraduate Research Experience (CURE) involving 16–20 students per year to identify amino acids crucial for NADase activity of *A. baumannii* TIR domain protein and the TIR from *Escherichia coli* (TIR domain-containing protein C). Students used structural data to identify amino acids that they hypothesized would play a role in TIR NADase activity, and created plasmids to express mutated TIRs through site-directed mutagenesis. Mutant TIRs were expressed, purified, and tested for NADase activity. The results from these studies provide evidence for a conformational change upon NAD binding, as was predicted by recent cryogenic electron microscopy and hydrogen-deuterium exchange mass spectrometry studies. Along with corroborating recent characterization of TIR NADases that could contribute to drug development for diseases associated with dysregulated TIR activity, this work also highlights the value of CURE-based projects for inclusion of a diverse group of students in authentic research experiences.

## Introduction

Contemporary undergraduate laboratory course design replaces traditional “cookbook-type” laboratory exercises with strategies such as Course-Based Undergraduate Research Experiences (CUREs) that integrate students into authentic research projects, often associated with faculty research programs (1, 2). Students who participate in CUREs report increased self-efficacy with experimental procedures and increased interest and enthusiasm toward scientific research (3, 4). CURE courses, as opposed to the traditional apprenticeship independent research model, have the potential to reach a broader range of students and have been identified as a strategic practice for increasing the diversity of undergraduate Science, Technology, Engineering and Mathematics (STEM) disciplines (5, 6). Besides expanding capacity for research training, CUREs use class time for research experiences, thus providing opportunities for students with outside obligations, including jobs, commutes, or family responsibilities (7, 8). In addition to these student-centered benefits, faculty members teaching CUREs also report benefits, including increased job satisfaction and creation of datasets that can propel faculty research programs, which is particularly useful for faculty at primarily undergraduate institutions, who often carry high teaching loads and limited research budgets (9).

We have developed a CURE course at Towson University (TU), a primarily undergraduate institution, to involve students in ongoing research related to the structure–function relationships of bacterial Toll/IL-1R (TIR) domain-containing proteins. Bacterial TIRs were first identified based on their homology to TIRs found in mammalian TLRs and their signaling adaptors (10). Given the central role that TLRs play in innate immune responses (11), bacterial pathogens that have evolved mechanisms to evade TLR responses would be anticipated to have a selective advantage in growth and survival within host organisms. Indeed, bacterial TIRs, including *Escherichia coli* and *Brucella* TIR domain-containing protein C (TcpC) and TcpB cross host cell membranes, bind with host TLRs, and block innate immune responses, leading to enhanced survival within host organisms (10, 12–14). Other bacterial TIRs, such as *Staphylococcus aureus* TirS, *Pseudomonas aeruginosa* PumA, *Salmonella* TcpS, *Enterococcus fecalis* TcpF, *Yersinia pestis* YpTdp, and *Yersinia ruckeri* STIR-2 also have been implicated in inhibition of TLR signaling (15–20).

In addition to direct interference with host innate immune signaling, several bacterial TIR domain-containing proteins also function to cleave NAD (21). The NADase activity of TIR domains was first characterized for the mammalian sterile α and TIR motif-containing 1 (SARM1) (22), and SARM1 NADase activity was shown to promote neuronal death by NAD^+^ depletion in cases of neuronal injury or infection (22, 23). The significance of NADase activity by bacterial TIRs for bacterial function and survival is not clear, and it is not known to what extent NADase activity plays a role in bacterial TIR interference of host innate immune responses. Recently, NADase activity of the bacterial TIR Thoeris B was found to be important for the bacterial antiphage defense system Thoeris (24–26), underlying the potential for involvement of bacterial TIR NADase enzymatic function in additional molecular processes that promote bacterial growth, survival, and virulence.

The prototypical TIR structure described by Xu et al. (27) is composed of five alternating α helical and β sheets (lettered A–E), in which the β sheets compose a central core surrounded by the α helices. Previous studies using X-ray crystallography and cryoelectron microscopy (cryo-EM) have characterized the structure of unbound *Acinetobacter baumannii* TIR domain protein (AbTir-TIR) and AbTir-TIR bound to an NAD^+^ analogue (28, 29). AbTir-TIR retains a prototypical TIR structure, with overall similarities to solved structures of other bacterial TIRs.

The NADase function of most bacterial TIRs depends on a conserved glutamate residue in the C-helix WxxxE motif. Interestingly, in the ligand-free AbTir-TIR crystal structures, the conserved glutamate (E208) is surface exposed and appears to be pointing away from the structure (28, 29). Several lines of evidence indicate that the ligand-free structure may represent an initial conformation, and that AbTir-TIR undergoes conformational changes upon ligand binding. First, cryo-EM structural determination of AbTir-TIR bound to an NAD^+^ analogue revealed the formation of a filamentous assembly of AbTir-TIR monomers that was accompanied by conformational changes in AbTir-TIR BB-loops and αB and αC helices (28). Site-directed mutagenesis of amino acid residues in these loops and helices verified the importance of these regions for NADase enzymatic activity (28). Hydrogen-deuterium exchange mass spectrometry (HDX-MS) analysis indicated that large conformational shifts occur in the unbound state, which appeared to stabilize upon addition of NAD^+^ (29).

In this article, we report results from the CURE course described earlier in which students used site-directed mutagenesis to further characterize amino acids critical for NADase activity of the bacterial TIRs, *A. baumannii* AbTir and *E. coli* TcpC. Mutational results provide additional evidence that the bacterial TIR NADase active site undergoes conformational changes upon NAD binding. Several amino acids found important for NADase activity are positioned oriented away from or located far from the catalytically important E208 residue in the unbound crystal structure, but near the dimer interface in the cryo-EM structure, where the ligand is proposed to sit (28, 29). These results reinforce previous evidence for a conformational change upon ligand binding and highlight the potential for data collected from CURE courses to contribute to timely scientific advances.

## Materials and Methods

### CURE course outline

An analysis of amino acids important for NADase function of the bacterial TIRs AbTir and TcpC was undertaken as a CURE course project in the TU Cell Biology Laboratory (Biol 412) in fall semesters from 2019 to 2022. Biol 412 is a three-credit upper-level undergraduate course with an enrollment of 16–20 students each semester. Completion of a cell/molecular-related CURE course, such as Biol 412, is a requirement for TU Biology majors with a cell/molecular concentration. Prerequisites for the course include the two-semester biology major introductory sequence: Biology 1: Introduction to Cellular Biology and Genetics with Laboratory (Biol 200/L) and Biology II: Introduction to Ecology and Evolution with Laboratory (Biol 206/L), as well as an intermediate course in genetics that does not include a wet laboratory (Biol 309). Students must also have completed General Chemistry 1 with Laboratory (Chem 131/L). The laboratories in the introductory courses at Towson include some inquiry-based exercises but are typically not taught as CURE courses. For most students in the course, the Cell Biology CURE course represents their first research-based laboratory experience; however, there are a subset of students in the course with previous independent research experience, either through summer research programs or independent research in faculty laboratories. The course includes 6 h of class time per week (one 2-h and one 4-h session) for 14 wk and is taught by a faculty instructor with support from one to two undergraduate learning assistants (ULAs) each semester.

**FIGURE 1.**
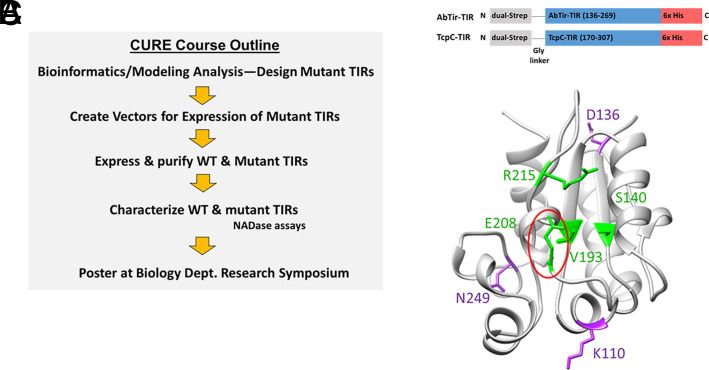
A CURE course was designed for the characterization of AbTir NADase activity. (**A**) CURE course outline. (**B**) Protein expression constructs contained the TIR domains from AbTir (aa 136–269) and TcpC (aa 170–307) flanked by an N-terminal dual-Strep tag and a C-terminal 6× his tag for purification. (**C**) Students in the CURE class analyzed the crystal structure of unbound AbTir (29). Shown are a subset of amino acids whose mutations were predicted to block NADase activity (labeled in green) or have no effect (labeled in purple) based on proximity to the previously identified catalytically important E208 residue (circled in red).

The overall outline of the course project is depicted in Fig. 1A, with specific weekly activities that reinforce course objectives listed in Table I. Initial weeks were devoted to practice of basic laboratory techniques (pipetting, laboratory math, dilutions, etc.), as well as journal club–based discussions of primary literature related to the project background before students embarked on the course project. Students then analyzed the structures of AbTir-TIR or the model of TcpC-TIR using the molecular visualization software UCSF Chimera (https://www.cgl.ucsf.edu/chimera/) and identified an amino acid that they predicted would be critical for TIR NADase activity (note that UCSF ChimeraX is the updated, supported version of UCSF Chimera but had not yet been fully developed during the initial CURE course offerings). Students then designed primers for PCR-based site-directed mutagenesis to create vectors encoding mutant TIRs (Supplemental Table I). Constructs encoding mutant TIRs with a StrepTag and HisTag were created in pET30 vectors and verified by Sanger sequencing. Proteins were expressed and purified using nickel beads, and NADase activity of the mutant TIRs was assessed using the EnzyChrom NAD/NADH assay, as described later. Students communicated their results to a broader scientific audience by presenting a poster outlining the results at the annual TU Fall Biology Department Symposium.

**Table I.** CURE schedule and objectives

Weeks	Laboratory Activities	Course Objectives
1–3	Journal clubs—bacterial TIR background papers Pipetting exercise Dilutions exercise Laboratory math practice problems	Read and discuss primary scientific literature. Describe the use of basic laboratory tools and equipment. Calculate dilutions, molar concentrations, and percent solutions required for preparing experimental buffers and reagents.
4–5	Chimera bioinformatics exercises Group PowerPoint presentation to discuss proposed mutation Primer design PCR-based site-directed mutagenesis	Analyze DNA sequences using basic bioinformatics techniques. Analyze protein structural models using molecular visualization software. Derive a scientific hypothesis. Communicate scientific data through an oral presentation. Design a strategy and use PCR-based site-directed mutagenesis to create a plasmid for mutant recombinant protein expression.
6–7	Bacterial transformation Isolation of plasmid DNA DNA electrophoresis DNA sequencing	Practice basic cell/molecular biological techniques. Analyze DNA sequencing results.
8–9	Small-scale recombinant protein expression Nickel bead purification SDS-PAGE Coomassie-based gel staining	Practice basic cell/molecular biological and biochemical techniques. Interpret results from purification of a recombinantly expressed protein.
10–12	NADase activity trials BCA protein quantification assays	Perform and interpret results from an NADase enzymatic assay. Perform and interpret results from a protein quantification assay. Perform biological repeats and quantitative analysis of results.
13–14	Prepare final poster presentations	Interpret and summarize results from a series of experiments. Communicate research findings to a broader scientific audience through a poster presentation.

Students were guided each week through the experimental process, with short lectures introducing the theories related to the laboratory techniques employed, and practical tips related to experimental methodology. In carrying out the experiments, students were divided into groups of four. For the bioinformatics analysis, each student in the group was responsible for identifying an amino acid that they anticipated might be important for NADase activity using bioinformatics tools and molecular visualization software. The students in each group, with input from the instructor, chose one of these amino acids for further analysis. Each student performed site-directed mutagenesis, transformation, and DNA extractions individually, so that at least four separate plasmids for each mutation could be analyzed by sequencing. In our experience, when analyzing four separate plasmids, we found in almost every case that at least one of those four plasmids exhibited the desired point mutation without any additional mutations. All subsequent experiments (protein expression, purification, and enzymatic analysis) were performed together by pairs of students so that each trial was performed in duplicate (one trial completed by each of the two pairs of students in the group of four). Due to the nature of these experimental procedures (i.e., protein purification involved multiple wash and elution steps; protein gel electrophoresis involved loading of noninduced and induced samples, as well as wild-type [WT] and E208 control samples), there were opportunities for students in the pairs to perform each of the steps of the experiment. In addition, the course schedule included at least one repeat of protein expression, purification, and enzymatic analysis experiments, providing additional opportunities for students to practice technical steps.

The course setup allowed 20 students in a semester to screen five mutations in two separate trials with duplicate samples in each trial. Following the CURE course, the results for all student-derived mutant proteins were verified by the course instructor, ULAs, or independent research students who had at least a semester’s previous experience conducting independent research in the instructor’s research laboratory.

### Protein expression and purification

The protein expression constructs encoding for the StrepTag-AbTir-TIR-HisTag and the StrepTag-TcpC-TIR-HisTag within the pET30a^+^ vector were obtained from Dr. K. Essuman (Fig. 1B) (21). Single amino acid substitution mutations were created by PCR-based site-directed mutagenesis using the AbTir-TIR^wt^ and TcpC-TIR^wt^ constructs as templates. Mutants were verified by Sanger sequencing and transformed into T7 Express *lysY* competent cells (New England Biolabs, Ipswich, MA). For expression, T7 Express cells were grown to log phase (OD_600_ 0.6–0.8) and induced for expression of AbTir-TIR and TcpC-TIR with 0.5 mM isopropyl-β-d-1-thiogalactopyranoside (IPTG) at 18°C for 6 h. Cell pellets were harvested and frozen at −80°C in 25 mM Tris-HCl (pH 7.4), 150 mM NaCl buffer. His-tagged TIR proteins were purified after sonication by nickel bead extraction (30). Protein expression was verified by elution with 0.2 M imidazole in 25 mM Tris-HCl (pH 7.4), 150 mM NaCl, and SDS-PAGE analysis of eluants, probing with GelCode Blue Safe Protein Stain (ThermoFisher Scientific, Waltham, MA).

### Measurement of NADase activity of proteins purified on nickel beads

T7 Express competent cells transformed with pet30^+^ vectors encoding for StrepTag-AbTir-TIR-HisTag WT and mutant constructs were induced for expression of AbTir-TIR with IPTG at 18°C for 6 h, harvested in 25 mM Tris-HCl (pH 7.4), 150 mM NaCl buffer and sonicated. Protein aggregates were removed from the samples by centrifugation at 17,000 × *g* for 10 min. His-tagged proteins were purified on nickel beads, and nickel beads were washed with 25 mM Tris-HCl (pH 7.4), 150 mM NaCl buffer and divided into three aliquots for NADase analysis, measurement of BCA levels, and verification of protein expression and purification by SDS-PAGE analysis.

For NADase analysis, nickel beads bound to AbTir-TIR were incubated with 5 µM NAD^+^, shaking at 22°C for 30 min. NAD levels remaining in samples were analyzed using the EnzyChrom NAD/NADH assay (BioAssay Systems, Hayward, CA) according to the manufacturer’s instructions and compared with the levels of NAD^+^ in samples incubated without protein.

To verify consistency of protein levels in AbTir-TIR samples, we eluted proteins from nickel beads with 0.2 M imidazole in 25 mM Tris-HCl (pH 7.4), 150 mM NaCl. Elutions were diluted to imidazole concentrations of 0.01 M, which was identified as an imidazole concentration that did not show interference with BCA measurements of protein samples. Protein levels of diluted eluants were measured using the BCA Protein Assay Kit (ThermoFisher Scientific), according to the manufacturer’s directions. Protein levels were normalized to those from WT samples.

### Measurement of NADase activity of purified proteins

T7 Express competent cells transformed with pET30^+^ vectors encoding for StrepTag-AbTir-TIR-HisTag WT and mutant constructs were induced for expression of AbTir-TIR with IPTG at 18°C for 6 h, harvested in 25 mM Tris-HCl, 150 mM NaCl buffer, frozen at −80°C, and sonicated. Protein aggregates were removed from the samples by centrifugation at 17,000 × *g* for 15 min. His-tagged AbTir-TIR proteins were purified by nickel bead chromatography, eluting with 0.2 M imidazole following a buffer and 0.02 M imidazole wash. Eluate samples were concentrated and subjected to buffer exchange with 25 mM Tris-HCl, 150 mM NaCl with Amicon ultra filters (10-kDa cutoff; Millipore Sigma, Burlington, MA). Protein expression and purification were verified by SDS-PAGE analysis.

For NADase analysis, 40 µg of purified AbTir-TIR proteins was incubated with 5 µM NAD^+^, shaking at 22°C for 60 min. NAD levels remaining in samples were analyzed using the EnzyChrom NAD/NADH assay (BioAssay Systems) according to the manufacturer’s instructions.

### Measurement of NAD levels in T7 Express competent cells expressing TcpC-TIR

T7 Express competent cells transformed with pET30^+^ vectors encoding for StrepTag-TcpC-TIR-HisTag WT and mutant constructs were induced with IPTG shaking at 18°C. After 6 h, OD_600_ of cultures was measured, and an equivalent of 1350 µl of cells at OD 1.0 was harvested, washed with ice-cold PBS, and frozen at −80°C in 200 µl of NAD extraction buffer, supplied with the EnzyChrom NAD/NADH assay. Samples were thawed, sonicated, heated at 60°C for 5 min, and added to 40 µl Assay Buffer and 100 µl NADH buffer supplied with the NAD/NADH assay. Samples were vortexed and centrifuged, and NAD levels in samples were measured using the EnzyChrom NAD/NADH assay kit, according to the manufacturer’s protocols.

### Statistical analysis

Data are presented as means ± SEM. Group mean differences were analyzed by one-way ANOVA, followed by Tukey’s post hoc analysis. Levels of significance were set at *p* < 0.05. Statistical analysis was performed using GraphPad Prism software (v. 10; GraphPad, Boston, MA).

### CURE student authorship

Attempts were made to contact all students enrolled in the Cell Biology Laboratory CURE course from fall 2019 to fall 2022 regarding authorship on the manuscript. A manuscript draft was sent to e-mail addresses provided to TU upon the students’ graduation and permanent e-mail addresses that had been provided by students upon completion of the course. CURE alumni who acknowledged receipt, read the manuscript, and approved its submission were listed as authors. To provide credit to students who did not respond to requests for manuscript approval but had been enrolled in the CURE and contributed to the study, we have included a list of all students enrolled in the CURE course from fall 2019 to fall 2022 in Supplemental Table II.

## Results

### A set of amino acids predicted to affect AbTir NADase activity was selected by students in a CURE course for characterization by site-directed mutagenesis

To better characterize the molecular requirements for NADase function, we set out to design a CURE course in which students carried out site-directed mutagenesis studies to characterize amino acid residues critical for NADase function (Table I). Students in the CURE course used UCSF Chimera to analyze the molecular structure of AbTir-TIR and to make predictions about amino acids that they hypothesized might affect AbTir-TIR NADase function. For this, students used our X-ray crystal structure of an apo AbTir-TIR domain (PDB code: 8G83) (29) that was unpublished and under refinement at the time of the CURE classes. In this unbound structure, the E208 residue, which previously had been identified as a key amino acid for NADase function (21), is located on the surface, oriented externally, indicating that a conformational change at the TIR active site might take place upon NAD binding (Fig. 1C). Recent cryo-EM and HDX-MS studies have provided experimental evidence for such a conformational change (28, 29). The cryo-EM structure (PDB code: 7UXU) had not yet been published at the time of the CURE course offerings, so the students’ analysis was restricted to the apo AbTir-TIR X-ray structure.

Considering the possibility that a conformational change might occur upon NAD binding, students chose amino acids to mutate based not only on proximity to the E208 residue in the unbound structure but also conservation of amino acids across bacterial TIRs and chemical properties of the amino acids they proposed to mutate (Fig. 1C, Table II). Mutants were created in the pET30-AbTir-TIR vector by site-directed mutagenesis. Mutant constructs were verified by sequencing and transformed into T7 Express *lysY E. coli* for expression.

**Table II.** Properties of AbTir-TIR amino acids selected by CURE students for mutational analysis

AbTir Amino Acid	NADase Activity	Loop Position	Proximity to E208 in Unbound Structure	Conservation in TcpC	NADase Activity of TcpC Mutant
D136A	No effect	βA	No	D172	Not tested
F138A	No expression	βA	Yes	F174	No effect
S140A	Inhibition	βA	Yes	S176	Inhibition
H141A	No effect	βA	Yes, but pointing away	H177	No effect
K146A	No effect	AA/αA	No	K182	No effect
V150A	No effect	αA	No	V186	Not tested
R151A	Inhibition	αA	No	R187	Inhibition
D167A	Inhibition	βB	No	D203	Inhibition
V193A	Inhibition	βC	Yes	V229	Inhibition
K201A	No effect	αC	No	Not conserved	Not applicable
W204A	Inhibition	CC	Yes	W240	No effect
E208E	Inhibition	αC'	E208	E244	Inhibition
R215A	Inhibition	CD	No	R251	Not tested
M222A	No effect	βD	Yes, but pointing away	Not conserved	Not applicable
Y238A	Inhibition	αD	Yes	Y275	No effect
N249A	No effect	βE	No	Not conserved	Not applicable

### Mutant recombinant AbTir-TIR proteins were successfully expressed and purified by students in a CURE course

**FIGURE 2.**
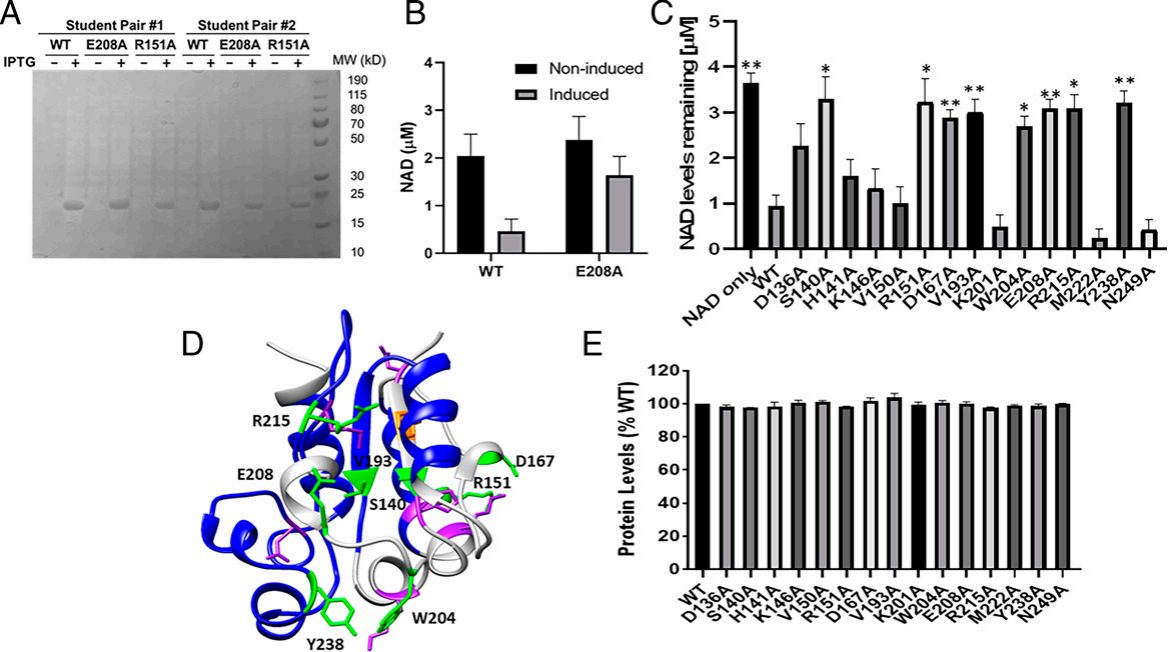
Mutational analysis of AbTir-TIR identifies amino acids critical for NADase activity. (**A**) SDS-PAGE analysis of *E. coli* lysates with or without 0.5 mM IPTG for induction of AbTir-TIR recombinant protein expression and purified with Ni-NTA. Results of a representative experiment are shown, performed by a group of four CURE students. Each group of four was divided into two pairs, in which each pair of students was responsible for purification of the AbTir-TIR^WT and E208A^ control proteins and their selected mutant protein (in this case, AbTir-TIR^R151A^). (**B**) NAD levels decreased upon incubation with AbTir-TIR^WT^. Nickel bead purifications from cultures with or without IPTG for expression of AbTir-TIR^WT and E208A^ were incubated with 5 µM NAD for 30 min at room temperature, and NAD levels were measured. Means and SEM from at least three biological replicates are presented. (**C**) NAD levels remaining following a 30-min incubation with AbTir-TIR mutant proteins purified on nickel beads. Means and SEM from at least three biological replicates for each mutation are presented and analyzed by one-way ANOVA (*p* < 0.0001), followed by Tukey’s post hoc analysis. Asterisks indicate statistically significant differences compared with AbTir-TIR^WT^ (*p < 0.01, **p < 0.0001). (**D**) Results of mutational analysis of AbTir-TIR NADase activity mapped onto the unbound crystal structure. Amino acids whose mutations inhibit NADase activity are shown in green, and those whose mutations do not affect NADase activity are shown in purple. AbTir-TIR mutation F138A (shown in orange) was not successfully expressed. Regions previously shown by HDX-MS analysis to undergo conformational change upon ligand binding are shown in blue. (**E**) Quantification of protein levels eluted from aliquots of nickel beads used for NADase analysis shown in (C). Relative protein levels eluted from nickel beads purifying mutant protein are calculated as the percentage of protein levels eluted from nickel beads purifying AbTir-TIR^WT^ proteins and presented as means and SEMs from at least three biological replicates for each mutation.

The pET30-AbTir-TIR vector uses an IPTG-inducible promoter and was constructed with an N-terminal streptavidin tag and C-terminal hexahistidine tag incorporated into the coding region to allow for facilitated protein purification. T7 Express *E. coli* cells transformed with pET30-AbTir-TIR vectors encoding for student-derived mutants were induced with IPTG for protein expression, expressed AbTir-TIR proteins were purified by nickel bead extraction, and purified protein was analyzed by SDS-PAGE and visualized by Coomassie staining. Data shown in Fig. 2A are representative of SDS-PAGE results from a group of students’ protein expression and purification experiments. Students worked in groups of four, with pairs completing duplicate trials to account for errors. All trials were replicated at least three times, with at least one trial by the instructor, ULAs, or independent research students who had at least a semester’s previous experience conducting independent research in the instructor’s research laboratory. Protein expression and purification were confirmed for all AbTir mutations made except in the case of AbTir F138A mutant (Table II).

### Analysis of mutations created in the CURE course identifies amino acids critical for NADase activity of AbTir

Once protein expression and purification were verified, mutant AbTir proteins were analyzed for NADase activity. Recombinant mutant proteins were expressed in *E. coli* upon IPTG induction and isolated on nickel beads as described. Proteins on nickel beads were incubated with 5 µM NAD for 30 min at 22°C. Levels of NAD remaining in the samples were analyzed using the Bioassay Enzychrom NAD/NADH Assay Kit. Incubation with WT AbTir-TIR proteins resulted in the loss of NAD in the samples, whereas NAD levels remained high in samples incubated with the NADase-inactivated AbTir-TIR E208A mutant, as well as in samples with nickel beads incubated with lysates from noninduced cells (Fig. 2B). The NADase activity assay was then performed with the mutated proteins designed, created, and expressed in the CURE courses, and results suggested that amino acid mutation affected the NADase activity of recombinant AbTir-TIR proteins (one-way ANOVA, *p* < 0.0001). In cases where the mutant protein retained activity, NAD levels decreased in the samples; whereas in cases where a loss of function was observed, NAD levels remained high (Fig. 2C, 2D). To ensure that protein levels on beads were consistent among the mutant AbTir-TIR proteins, we measured protein levels eluted from an aliquot of the nickel beads used to purify AbTir-TIR proteins using a BCA assay. Protein levels were found to be consistent across the AbTir-TIR mutant purifications used for the NADase trials (Fig. 2E). As with the earlier protein expression and purification trials, pairs of students replicated results for each mutant protein, and all results were further verified by the instructor or experienced ULAs.

**FIGURE 3.**
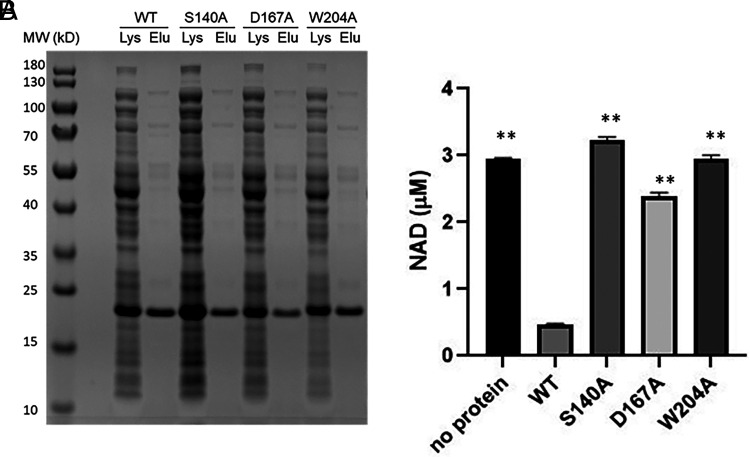
Analysis of NADase activity of AbTir-TIR proteins purified by nickel bead chromatography. (**A**) SDS-PAGE analysis of recombinant AbTir-TIR^WT, S140A, D167A, and W204A^ proteins expressed in lysates (lys) and purified and eluted (Elu) from a Ni-NTA column with 0.2 M imidazole. (**B**) Analysis of NADase activity of purified AbTir-TIR proteins. Purified AbTir-TIR proteins were incubated with 5 µM NAD for 1 h at room temperature, and remaining NAD levels in the sample were measured. Means and SDs from three replicates are presented and analyzed by one-way ANOVA (*p* < 0.0001), followed by Tukey’s post hoc analysis. Asterisks indicate statistically significant differences compared with NAD levels remaining upon incubation with Ab-TIR^WT^ (**p < 0.0001).

Following completion of the CURE course, a subset of mutant AbTir-TIR proteins (S140A, D167A, and W204A), as well as WT AbTir-TIR, was recombinantly expressed and purified by nickel bead chromatography (Fig. 3A). The purified recombinant proteins were incubated with 5 µM NAD and tested for the loss of NAD in the samples. Corroborating results from Fig. 2C, whereas incubation with WT AbTir-TIR purified recombinant protein resulted in loss of NAD in the samples, NAD levels remained high in samples incubated with purified AbTir-TIR mutants S140A, D167A, and W204A (Fig. 3B).

**FIGURE 4.**
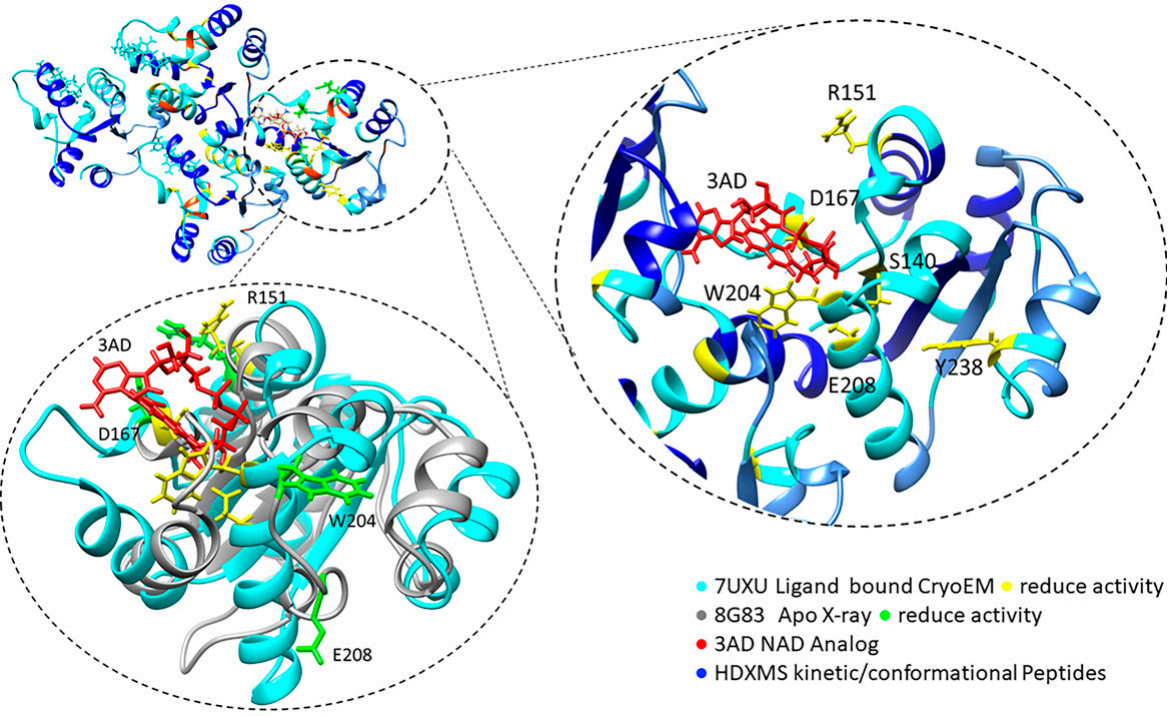
A subset of amino acids identified to be important for NADase activity is located near the ligand in the cryo-EM structure of ligand-bound AbTir. Modeling of the amino acids mutated in this study on the ligand-bound cryo-EM structure of AbTir (28). The upper right insert depicts a magnification of the region surrounding the ligand (shown in red) in the cryo-EM structure. Amino acids near the ligand whose mutations inhibit NADase activity are shown in yellow. As in Fig. 2C, regions previously shown by HDX-MS to undergo conformational change upon ligand binding are shown in blue. The lower right insert depicts the same region from the cryo-EM structure compared with the unbound AbTir-TIR X-ray crystal structure (29), with amino acids whose mutations affect NADase activity shown in green. Comparison between the two structures shows repositioning of secondary structures and loops, including those that contain aa D167, W204, and E208, all found to be critical for AbTir NADase activity.

Results summarizing the NADase activity for each mutant protein tested in the CURE course are shown in Fig. 2D and Table II. Students found that for many of the mutations, the observed NADase activity was as they had anticipated, with several of the mutations in amino acids found in the vicinity of the catalytically important E208 residue in the unbound crystal structure, such as S140, V103, W204, R215, and Y238, showing statistically significant reduction in NADase activity (Fig. 2C, 2D). A subset of the amino acid mutations, however, showed results different from those predicted based on analysis of the crystal structure. Notably, amino acid mutations at D167 and R151 inhibited NADase activity in a statistically significant manner, even though both of these amino acids are positioned relatively far from the E208 residue in the unbound crystal structure (Fig. 2C, 2D). The amino acid R151 is localized to a region of AbTir-TIR that has been shown recently using HDX-MS studies to exhibit large conformational shifts upon ligand binding (Figs. 2D, 4) (29). Further modeling indicates that the D167 residue appears to show interactions with the ligand analogue in the recently published analogue ligand-bound cryo-EM structure (28), which shows a conformational shift compared with the unbound crystal structures (Fig. 4).

### Mutational analysis of TcpC reinforces findings from AbTir regarding amino acids critical for NADase activity of bacterial TIRs

**FIGURE 5.**
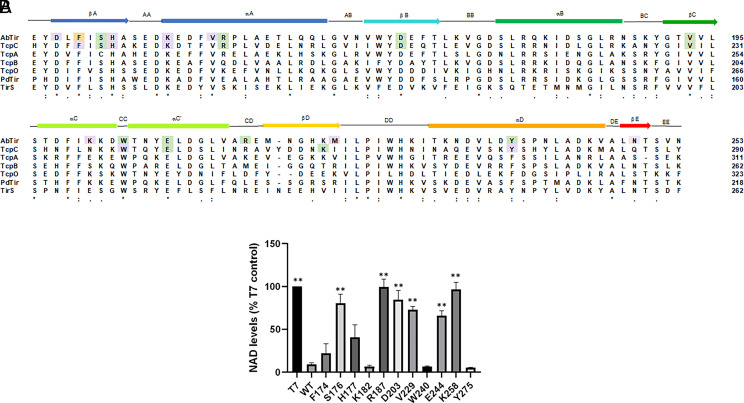
Mutational analysis of the NADase activity of amino acids conserved in TcpC reinforces findings from AbTir-TIR mutational analysis. (**A**) Sequence alignment of AbTir-TIR with TcpC-TIR and additional bacterial TIRs previously found to exhibit NADase activity using Clustal Omega (https://www.ebi.ac.uk/jdispatcher/msa/clustalo). Amino acids whose mutations are shown here to affect NADase activity are highlighted in green, whereas those whose mutations do not affect NADase activity are shown in purple. Expression and purification of AbTir-TIR mutated at F138 (highlighted in orange) was not successful. Asterisks (*) indicate conserved residue; double vertical dots (:) indicate conservation of amino acids with strongly similar properties; single dots (.) indicate conservation of amino acids with weakly similar properties. (**B**) NAD levels of *E. coli* lysates harvested 6 h after IPTG induction of expression of WT and mutant TcpC-TIR recombinant proteins were measured. Means and SEM from at least three biological replicates for each mutation are presented and analyzed by one-way ANOVA (*p* < 0.0001), followed by Tukey’s post hoc analysis. Asterisks indicate statistically significant differences compared with levels of NAD found in *E. coli*–expressing TcpC-TIR^WT^ (**p < 0.0001).

Students’ choices of AbTir mutations to analyze were influenced by the level of conservation of amino acids among other bacterial TIR proteins. For one semester’s CURE class, instead of creating AbTir mutations, students identified conserved amino acids in the *E. coli* TIR protein TcpC that they hypothesized would affect TcpC NADase function (Fig. 5A). As with AbTir mutations, pET30 vectors encoding the *E. coli* TcpC TIR domain with an N-terminal streptavidin tag and C-terminal hexahistidine tag were used as templates for PCR-based site-directed mutagenesis. Mutants were verified by Sanger sequencing and transformed into T7 lysY *E. coli* for expression upon IPTG induction. Protein expression was confirmed by SDS-PAGE analysis of His-tagged TcpC-TIR proteins purified on nickel beads (data not shown). To analyze NADase activity of mutant TcpC-TIR proteins, we analyzed NAD levels in lysates of *E. coli* expressing the recombinant TcpC-TIR proteins using the Bioassay Enzychrom NAD/NADH assay kit. Expression of WT TcpC-TIR resulted in significantly lower levels of NAD in *E. coli* lysates than expression of TcpC-TIR^E244A^, which contains a mutation in a glutamic acid previously shown to be critical for activity of NADase hydrolases, including TcpC (Fig. 5B) (21). The CURE course in which students studied TcpC mutations was taught online during fall of 2020 because of COVID-19 pandemic social-distancing protocols, so all NADase experimental trials were completed by the instructor and/or experienced ULAs, with students in the CURE course using class time to plan experimental setup and analyze experimental results.

For most TcpC-TIR mutations analyzed, the NADase activity was consistent with that seen for the AbTir-TIR mutations (Fig. 5, Table II). If a mutation in AbTir resulted in loss of NADase activity, the corresponding mutation in TcpC also resulted in statistically significant loss of NADase activity. Exceptions were observed for W240 and Y275 in TcpC (W204 and Y238 in AbTir). Both of these conserved mutations in AbTir-TIR caused a loss of NADase activity, but activity remained high in corresponding mutant TcpC-TIR proteins.

### The CURE course engaged a diverse group of students in an authentic undergraduate research experience

**FIGURE 6.**
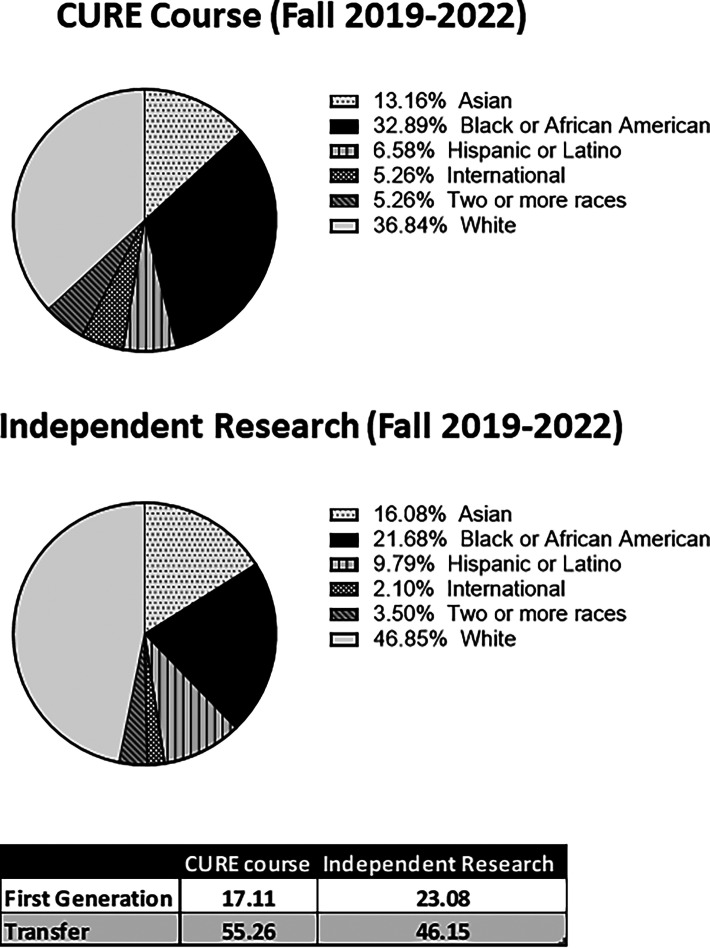
The CURE class engages a diverse group of students in undergraduate research. Comparison of the racial and ethnic diversity and number of first-generation and transfer students enrolled in the TU Cell Biology Laboratory CURE class compared with that of students enrolled in independent research courses in the TU Biology department and Molecular Biology, Biochemistry and Molecular Biology program between fall 2019 and fall 2022 (data sources are TU Office of Institutional Research compiled from Peoplesoft, Office of Institutional Research Enrollment Extracts, and Free Application for Federal Student Aid reports).

The Cell Biology Laboratory (BIOL 412) CURE course engaged 75 undergraduate students between 2019 and 2022. Of these students, >63% were students who identified as being from backgrounds other than non-Hispanic white, and 39.5% were from ethnic and racial backgrounds underrepresented in STEM (African American, Black, or Hispanic) (Fig. 6). By comparison, an analysis of students enrolled in independent research courses in the Biology and Molecular Biology, Biochemistry and Bioinformatics programs during the same time period revealed that 53% were students who identified from backgrounds other than non-Hispanic white and 31.5% were students from African American, Black, or Hispanic backgrounds (Fig. 6). Although a slightly higher percentage of students participating in independent research identified as first-generation students (23.08% versus 17.11% in the CURE class), a higher percentage of the students in the CURE class were transfer students (55.26% as compared with 46.15% in independent research) (Fig. 6). Graduating Senior Survey results from 2020–2022 indicated that 43% of respondents from the CURE course planned to pursue postgraduate work following graduation with their B.S. or B.A. degrees (data sources were TU Office of Institutional Research compiled from Peoplesoft, Office of Institutional Research Enrollment Extracts, and Free Application for Federal Student Aid reports).

## Discussion

In this article, we demonstrate the application of a CURE course for structural and functional characterization of bacterial TIR proteins. Using data from site-directed mutagenesis studies, students in the CURE class identified amino acids in the bacterial TIRs AbTir and TcpC that they predicted would play a role in NADase activity, and carried out studies to test their hypotheses. Although many of the amino acid mutations tested showed NADase activity results that would have been anticipated based on their vicinity to the catalytically important E208 residue in the AbTir-TIR unbound crystal structure, two of the amino acids in AbTir found distally from E208 (D167 and R151) were found to be critical for NADase activity. Notably, although the amino acid D167 is far from the E208 residue in the unbound AbTir-TIR crystal structure, in the ligand-bound cryo-EM structure, it is adjacent to E208 and in a position that suggests binding with the analogue ligand (Figs. 2C, 3) (28). The amino acid R151 is located in a region of AbTir that was identified through HDX-MS studies to show conformational change upon ligand binding (Figs. 2C, 3) (29). Further, in the cryo-EM structure, R151 is on the protein’s surface oriented toward the analogue ligand (Fig. 3). These data support recent findings from cryo-EM and HDX-MS studies that suggest that AbTir undergoes conformational changes upon NAD^+^ binding (28, 29).

Our studies extending mutant characterization to conserved amino acids in TcpC further reinforce the importance of the amino acids identified in the CURE class for bacterial NADase function. Most of the mutants tested in TcpC-TIR that were conserved in AbTir-TIR NADase activity showed similar NADase activity, including R187 and D203 (conserved with R151 and D167 in AbTir) being required for NADase activity by TcpC-TIR. There were two notable exceptions in the TcpC-TIR amino acids that we tested. The amino acids W240 and Y275 were not found to be necessary for TcpC-TIR, whereas the conserved amino acids W204 and Y238 were essential for AbTir-TIR NADase activity. Notably, Manik et al. (28) found that W204 in AbTir is required for production of the cyclic ADP ribose (cADPR) isomer 2′cADPR upon cleavage of NAD^+^. cADPR products of NAD cleavage by bacterial and plant TIRs have been shown to play various roles in cellular signaling (24, 28, 31, 32). Manik et al. (28) found that the tryptophan residue is strongly conserved among TIR domains that produce a cADPR product but weaker among noncatalytic TIRs or TIRs that produce non-cADPR products from NAD^+^ cleavage. Although the tryptophan residue is conserved in TcpC, cleavage of NAD^+^ by TcpC results in production of the noncyclic version of ADPR (21). The Y238 residue in AbTir has not been characterized previously but is positioned facing W204 in the unbound AbTir-TIR crystal structure (Fig. 2C) (29). Further investigation is needed to determine whether the lack of requirement for W240 and/or Y275 for TcpC NADase activity might be related to the lack of production of cADPR upon NAD cleavage by TcpC.

It should be noted that TIR domain-containing proteins possess oligomerization motifs that promote TIR–TIR associations, and that the group of TIR domain-containing proteins with NADase activity undergo self-association to facilitate their enzymatic activity (33). These self-associations have been better characterized for mammalian SARM1 (23, 34) and plant TIR domains (35) than for the bacterial and archaeal TIR domains. Recent structural characterization of AbTir-TIR (28, 29) indicates that filamentous assembly of AbTir-TIR domain dimers and higher-order oligomerization accompanies enzymatic activity. It remains to be determined whether the loss of NADase activity by each of the mutant AbTir-TIR proteins analyzed in this study might be attributed to alterations in higher-order oligomerization of the mutated TIR proteins.

The benefits of CURE courses are often discussed in terms of the value provided for students in promoting student scientific skills, science identity, self-efficacy, and persistence in STEM (1, 3–5, 8, 36, 37). Although these benefits are significant and well documented in the literature, the outcomes of the CURE course in this study provide an example of an additional goal of a CURE class: generating data that contribute to ongoing studies in a broader research field, in this case, structural and functional studies of bacterial TIRs. This highlights an important benefit of CURE classes in promoting faculty research programs that is particularly acute for faculty at predominantly undergraduate institutions, who tend to carry heavy teaching and advising loads. In a comprehensive survey of such faculty, >80% identified factors contributing to lack of time as major barriers to research productivity at their universities (38, 39). Adding to this is the reality that engagement of undergraduate students in research does not automatically equate with increased faculty scholarly productivity, which is one of the measures that correlates best with faculty pay and promotion across all institutions, regardless of Carnegie classifications (40). Faculty surveys have highlighted tips and strategies to efficiently engage undergraduate student researchers in research projects in ways that are most likely to lead to research publications (39, 41). One of the most potent strategies involves scheduling faculty time so that activities fulfill multiple obligations (39). For example, faculty teaching CURE courses contribute to their teaching loads while engaging in research activities that can lead to scholarly output (41–45). This synergy occurs not just in the hours spent face-to-face with students in the laboratory sessions but also in faculty time devoted to planning and preparing class activities and laboratory materials that doubles as time for reading background literature, analyzing experimental data, and thinking about research goals.

The course structure in this study may serve as a model for other faculty developing CURE courses looking to gain such benefits for themselves and for their students. A similar course structure involving site-directed mutational analysis could be adopted for other projects that characterize a protein’s function, provided a relatively straightforward assay can be applied for functional analysis. In vitro enzymatic analysis can be used, for example, with recombinant proteins for which protein expression and purification methods have been optimized, as was the case for the AbTir-TIR protein. Alternatively, site-directed mutational studies also could be used to characterize processes affected by transformation or transfection of plasmids encoding for mutated gene products in bacteria or cultured mammalian cells. Although the studies used this study coupled structural data derived from X-ray crystallography to develop the models students used to make predictions about amino acids important for protein function, with the recent development of AlphaFold (46), CURE projects can be developed in which students generate models for protein structure even in the absence of experimentally derived structural data.

This course was taught from 2019 to 2022, including semesters before, during, and after online learning related to the COVID pandemic. For the online version of the course taught in fall of 2020, students designed and interpreted experiments that were then performed by the instructor and ULAs. Students had access to recorded videos of the instructor or ULAs carrying out the techniques. To better engage students with the project aims during the online course, students worked with their groups in breakout rooms during mandatory synchronous sessions to complete structured assignments related to experimental design and data analysis. This resulted in a larger percentage of overall class time devoted to experimental planning, interpretation, and analysis activities. Furthermore, these types of activities took place to a greater extent throughout the full semester, rather than being concentrated near the end of the semester while students were preparing their final presentations. Upon return to in-person learning in fall of 2021, we made slight modifications to the course outline compared with that from before the pandemic. We dropped a module on Western blots to allow time for incorporation of the structured experimental design and data analysis exercises we had created during the pandemic. Although students in the course postpandemic tended to have less prior experience with hands-on laboratory techniques than those prepandemic, we did not need to make major adjustments in the course structure to accommodate for differences in learning curves for students prepandemic versus postpandemic. This may be because of a course structure that already devoted several early class sessions to practicing basic laboratory techniques before embarking on the course project, or potentially because the instructor’s previous experiences teaching the course fostered practices that promoted a more efficient development of these laboratory skills. Additional teaching materials for the CURE course that might assist faculty in creating a course modeled after this course structure, including the structured experimental design and data analysis exercises created during the online iteration of the course, are available at CUREnet (https://serc.carleton.edu/curenet/collection/277391.html).

Lastly, a demographic analysis of students in the CURE course described in this article indicated that there was an increase in the percentage of students who identified as members of ethnic and racial groups underrepresented in science as compared with the percentage of students completing individual research in faculty members’ laboratories in the Biology and Molecular Biology, Biochemistry and Bioinformatics programs at TU (39.5% versus 31.5%). In addition, transfer students made up a greater percentage of students in the CURE class than in independent research courses (55% versus 46%). It is projected that by the year 2043, individuals who identify as African American/Black, Indigenous, or Hispanic will make up >50% of the U.S. population. Given these anticipated demographic changes, the ability to grow and maintain a strong STEM workforce will require the education and training of a diverse group of scientists. In 2021, only 26% of bachelor’s degrees and 16% of doctoral degrees in STEM were awarded to students from these groups (47). The benefits of a diverse STEM workforce go beyond national demographics, because studies have shown that groups made up of individuals from diverse backgrounds are more efficient in developing innovative solutions to complex problems (48). Taken together, these findings highlight the importance of creating inclusive solutions for developing and promoting scientists from diverse backgrounds. CURE courses represent such a strategy, because they allow faculty to engage a large, diverse group of students in their research. This population includes those who often have been excluded from traditional apprenticeship research models because of factors including academic background, commitments outside of class to jobs or family, and unfamiliarity with academic research practices (5, 7, 49).

In summary, this study describes findings from a CURE course involving a diverse group of students who contribute to timely research on the structure and activity of bacterial NAD-hydrolase TIR proteins. The results in this article corroborate previous findings that the bacterial AbTir appears to undergo conformational changes upon ligand binding and raise questions about structural requirements that might differ for TIRs depending on NAD cleavage products formed. Furthermore, the results reinforce the concept that, as well as providing benefits for students, CURE courses also contribute to faculty scholarship and ongoing research in a scientific field.

## Supplementary Material

Supplemental Material (PDF)
