# Supplementary material for: Characterization of the Structural Requirements for the NADase Activity of Bacterial Toll/IL-1R domains in a Course-based Undergraduate Research Experience
Source: Immunohorizons. 2024 Aug 22;8(8):563–76. doi: 10.4049/immunohorizons.2300062 (PMC11374754; doi:10.4049/immunohorizons.2300062)
Supplement: Supplemental Material (PDF) [file IH_2300062_Supplemental_3.pdf]

Supplemental Table I: Site-Directed Mutagenesis Primers Used in this Study

Mutation	Primers (forward and reverse complement)
AbTir D136A	5'-GCGGTGCTAGCGAATATGCTCTGTTTATTAGCCATGCGAG<br>5'-CTCGCATGGCTAATAAACAGAGCATATTCGCTAGCACCGC
AbTir F138A	5'-GTGCTAGCGAATATGATCTGGCTATTAGCCATGCGAGCGAAG<br>5'-CTTCGCTCGCATGGCTAATAGCCAGATCATATTCGCTAGCAC
AbTir S140A	5'-CGAATATGATCTGTTTATTGCCCATGCGAGCGAAGATAAAG<br>5'-CTTTATCTTCGCTCGCATGGGCAATAAACAGATCATATTCG
AbTir H141A	5'-CGAATATGATCTGTTTATTAGCGCTGCGAGCGAAGATAAAGAAG<br>5'-CTTCTTTATCTTCGCTCGCAGCGCTAATAAACAGATCATATTCG
AbTir K146A	5'-CCATGCGAGCGAAGATGCAGAAGATTTTGTGCGC<br>5'-GCGCACAAAATCTTCTGCATCTTCGCTCGCATGG
AbTir V150A	5'-GAAGATAAAGAAGATTTTGCGCGCCCGCTGGCGGAAACC<br>5'-GGTTTCCGCCAGCGGGCGCGCAAAATCTTCTTTATCTTC
AbTir R151A	5'-GAAGATAAAGAAGATTTTGTGGCCCCGCTGGCGGAAACCCTGC<br>5'-GCAGGGTTTCCGCCAGCGGGGCCACAAAATCTTCTTTATCTTC
AbTir D167A	5'-GGCGTGAACGTGTGGTATGCTGAATTTACCCTGAAAGTGG<br>5'-CCACTTTCAGGGTAAATTCAGCATACCACACGTTCACGCC
AbTir V193A	5'-CAGCAAATATGGCACCGCGGTGCTGAGCACCG<br>5'-CGGTGCTCAGCACCGCGGTGCCATATTTGCTG
AbTir K201A	5'-GCTGAGCACCGATTTTATTGCAAAAGATTGGACCAACTATG<br>5'-CATAGTTGGTCCAATCTTTTGCAATAAAATCGGTGCTCAGC
AbTir W204A	5'-CCGATTTTATTAAAAAAGATGCGACCAACTATGAACTGGATGG<br>5'-CCATCCAGTTCATAGTTGGTCGCATCTTTTTTAATAAAATCGG
AbTir E208A	5'-TTTATTAAAAAAGATTGGACCAACTATGCACTGGATGGCCTGGTGGCGCGCGAAATGAAC<br>5'-GTTCATTTCGCGCGCCACCAGGCCATCCAGTGCATAGTTGGTCCAATCTTTTTTAATAAA
AbTir R215A	5'-CTGGATGGCCTGGTGGCGGCCGAAATGAACGGCCATAAAATG<br>5'-CATTTTATGGCCGTTCATTTCGGCCGCCACCAGGCCATCCAG
AbTir M222A	5'-GCGAAATGAACGGCCATAAAGCGATTCTGCCGATTTGGC<br>5'-GCCAAATCGGCAGAATCGCTTTATGGCCGTTCATTTCGC
AbTir Y238A	5'-CAAAAACGATGTGCTGGATGCTAGCCCGAACCTGGCGGATAAAG<br>5'-CTTTATCCGCCAGGTTCGGGCTAGCATCCAGCACATCGTTTTTG
AbTir N249A	5'-GGCGGATAAAGTGGCGCTGGCCACCAGCGTGAACAGCATTG<br>5'-CAATGCTGTTCACGCTGGTGGCCAGCGCCACTTTATCCGCC
TcpC F174A	5'-GTGCTAGCCATTACGACTTCGCCATATCACATGCCAAAGAAG<br>5'-CTTCTTTGGCATGTGATATGGCGAAGTCGTAATGGCTAGCAC
TcpC S176A	5'-GCCATTACGACTTCTTCATAGCACATGCCAAAGAAGATAAG<br>5'-CTTATCTTCTTTGGCATGTGCTATGAAGAAGTCGTAATGGC
TcpC H177A	5'-CATTACGACTTCTTCATATCAGCTGCCAAAGAAGATAAGGACAC<br>5'-GTGTCCTTATCTTCTTTGGCAGCTGATATGAAGAAGTCGTAATG
TcpC K182A	5'-CATATCACATGCCAAAGAAGATGCGGACACATTCGTTCGCCC<br>5'-GGGCGAACGAATGTGTCCGCATCTTCTTTGGCATGTGATATG
TcpC R187A	5'-GAAGATAAGGACACATTCGTTGCACCCTTGGTCGACGAATTAAATC<br>5'-GATTTAATTCGTCGACCAAGGGTGCAACGAATGTGTCCTTATCTTC
TcpC D203A	5'-GGCGTCATAATCTGGTACGCTGAGCAGACGCTGGAAGTAG<br>5'-CTACTTCCAGCGTCTGCTCAGCGTACCAGATTATGACGCC

TcpC V229A	5'-GAAAGCAAATTATGGCATCGCAATCCTGTCCCACAACTTC<br>5'-GAAGTTGTGGGACAGGATTGCGATGCCATAATTTGCTTTC
TcpC W240A	5'-CCACAACTTCTTAAATAAGAAAGCGACCCAGTATGAGTTGGATTCGC<br>5'-GCGAATCCAACTCATACTGGGTCGCTTTCTTATTTAAGAAGTTGTGG
TcpC E244A	5'-CTTAAATAAGAAATGGACCCAGTATGCATTGGATTCGCTTATTAACCGTGCGGTTTAC<br>5'-GTAAACCGCACGGTTAATAAGCGAATCCAATGCATACTGGGTCCATTTCTTATTTAAG
TcpC K258A	5'-GCGGTTTACGATGACAATGCAATCATATTACCTATCTGGCATAAC<br>5'-GTTATGCCAGATAGGTAATATGATTGCATTGTCATCGTAAACCGC
TcpC Y275A	5'-CGCACAAGAAGTGAGTAAGGCCTCTCACTACCTGGCAGAC<br>5'-GTCTGCCAGGTAGTGAGAGGCCTTACTCACTTCTTGTGCG

Supplemental Table II: Students enrolled in the CURE course 2019-2022

Alexander,Zachary	David, Jaira	Riedlbauer,Dalton
Calkins,Phoebe	De Leon,Raquel	Schwartz,Jonathan
Chaudhry,Sumra	DeLoach,Kayla	Stefano,Alanna
Chomiak,Emily	Do,Aaron	Walsh,Kelly
Clark,Kishore	Eromosele,Itohan	Williams,Alexa
Conteh,Maimunah	Hillman,Jake	Akindileni,Kehinde
Dada,Tope	Madison Jr,Robert	Armstrong,Chyna
Fallah, Daniel	Melo-Gomez,Juliana	Bader,Charlotte
Hamilton,Sarah	Morales,Nadia	Biswas,Aditi
Jiang,Evelyn	Owolabi,Olutobi	Budd,Stephen
Jones,Brandon	Papageorgopoulos,William	Collins,Haley
Keshmiri,Nastaran	Pascoe,Emily	Duff,Rachel
Mark,Ashley	Sadowski,Lauren	Elsayed,Sarah
Moody,Jack	Shrestha,Amulya	Guadagno,Maria
Ojo,Michael	Suissa,Chad	Jean Baptiste,Henri
Poteat,Ariana	Anderson,Jasmine	Jones,Jaliyl
Riaz,Tahreem	Atkinson,Sy'mone	Kalu,Kelechi
Rolle,Louis	Badejo,Zainab	Langhorn,Trevor
Smith,Meeko	Campbell III,Michael	Onwezi-Nwugwo,Jaine
So,Nathan	Essilfie,Marina	Scudder,Alexis
Adeoye,Joycelyn	Evangelista,Justin	Shoemaker,Payton
Akin Abu,Gold	Mercanti,Mark	Sterling,Michaela
Akindele,Mercy	Njoroge,Wambui	Sultana,Samina
Akinwale,Oluwatobi	Portales Rivera,Alfredo	Toneygay,Lindsey
Darbasie,Brianna	Price,Sarah	Wiles,Monica
